# Histone Deacetylase Inhibitors Restore Cell Surface Expression of the Coxsackie Adenovirus Receptor and Enhance CMV Promoter Activity in Castration-Resistant Prostate Cancer Cells

**DOI:** 10.1155/2012/137163

**Published:** 2012-01-12

**Authors:** Laura Kasman, Georgiana Onicescu, Christina Voelkel-Johnson

**Affiliations:** Department of Microbiology & Immunology, Medical University of South Carolina, P.O. Box 250504, 173 Ashley Avenue, Charleston, SC 29403, USA

## Abstract

Adenoviral gene therapy using the death receptor ligand TRAIL as the therapeutic transgene can be safely administered via intraprostatic injection but has not been evaluated for efficacy in patients. Here we investigated the efficacy of adenoviral TRAIL gene therapy in a model of castration resistant prostate cancer and found that intratumoral injections can significantly delay tumor growth but cannot eliminate established lesions. We hypothesized that an underlying cause is inefficient adenoviral delivery. Using the LNCaP progression model of prostate cancer we show that surface CAR expression decreases with increasing tumorigenicity and that castration resistant C4-2b cells were more difficult to transduce with adenovirus than castration sensitive LNCaP cells. Many genes, including CAR, are epigenetically silenced during transformation but a new class of chemotherapeutic agents, known as histone deacetylase inhibitors (HDACi), can reverse this process. We demonstrate that HDACi restore CAR expression and infectivity in C4-2b cells and enhance caspase activation in response to infection with a TRAIL adenovirus. We also show that in cells with high surface CAR expression, HDACi further enhance transgene expression from the CMV promoter. Thus HDACi have multiple beneficial effects, which may enhance not only viral but also non-viral gene therapy of castration resistant prostate cancer.

## 1. Introduction

Epigenetic alterations, such as aberrant activity of histone deacetylases, are frequently observed in malignancies. Acetylation of histones is associated with less condensed chromatin and a transcriptionally active gene status, while deacetylation is associated with transcriptional silencing. Histone deacetylase inhibitors (HDACiS) were originally found to reverse the malignant phenotype of transformed cells and have subsequently been developed as a new group of chemotherapeutic agents. HDACi can affect numerous signaling pathways to inhibit growth or angiogenesis and induce apoptosis or senescence [1, 2]. Using two HDACi under evaluation for the treatment of prostate cancer, we previously demonstrated that both romidepsin (also known as depsipeptide) and MS-275 enhanced the *in vitro* efficacy of adenoviral TRAIL gene therapy in castration-sensitive LNCaP prostate cancer cells [3]. This effect was selective for the malignant cells as primary cultures of prostate epithelial cells were not adversely affected [3].

 TRAIL gene therapy has been evaluated for safety in prostate cancer patients with locally confined disease scheduled for prostatectomy [4]. Although efficacy was not evaluated as part of the trial, evidence of TUNEL staining in prostates injected with AdTRAIL indicated that DNA fragmentation, a hallmark of apoptosis, had occurred [4]. While these results are promising, novel therapies are still desperately needed for patients with aggressive disease. We therefore set out to evaluate the efficacy of TRAIL gene therapy against the aggressive, castration-resistant LNCaP-derived C4-2b cell line and to investigate potential benefits of therapy with HDACi. Our results suggest that HDACi have multiple effects that can be beneficial for the treatment of aggressive prostate cancers, particularly when used in combination with TRAIL gene therapy.

## 2. Materials and Methods

### 2.1. Cell Culture

LNCaP and its sublines C4-2 and C4-2b were grown in T-medium containing 5% heat-inactivated FBS and supplements [5], while 22Rv1 cells were cultured in RPMI1640 supplemented with 10% heat-inactivated FBS and nonessential amino acids. Heat-inactivated FBS was purchased from Hyclone, Logan, UT. Cell cultures were maintained at 37°C in a 5% CO_2_ atmosphere and were cultured in Primaria plasticware (Falcon, Bedford, MA).

### 2.2. Histone Deacetylase Inhibitors

MS-275 was purchased from Calbiochem (San Diego, CA). The Romidepsin was generously provided by Celgene Corporation and the National Cancer Institute, NIH.

### 2.3. Adenoviral Transduction

The adenoviral vectors have previously been described [3, 6]. Constructs were titered by counting GFP-positive cells, and titers were expressed as infectious units/mL (IU/mL). Cells were plated at 2 × 10^5^/well in 12-well plates. The next day cells were infected in growth medium at the indicated IU/cell. HDACi, if any, were given simultaneously. Fluorescence microscopy was performed with a Zeiss Axioscope 200 equipped with an MR5 camera.

### 2.4. Caspase-3/7 Assay

Cells were plated at 1 × 10^4^/well in a 96-well plate on the day prior to treatment initiation. HDACi, if any, were given simultaneously. The Apo-ONE Homogeneous Caspase 3/7 Assay (Promega, Madison, WI) was performed 24 hours after infection/treatment initiation according to the manufacturer's instructions. Plates were read using a FluoStar plate reader (BMG Labtechnologies Inc., Durham, NC) with emission and excitation wavelengths of 485 nm and 520 nm, respectively.

### 2.5. Transfection

Cells were seeded at 6 × 10^4^/well on a 24-well plate and allowed to attach overnight. Transfections were performed with Lipofectamine according to the manufacturers instructions using 0.4 *μ*g DNA and 2 *μ*L transfection reagent/well. The CMV-luciferase plasmid (pCMV.luc) was kindly provided by Dr. Omar Moussa, MUSC. Bioluminescence was quantified 48 hours after transfection using an IVIS Xenogen 200.

### 2.6. Flow Cytometry

For GFP analysis, cells were trypsinized, pelleted, and resuspended in PBS prior to flow cytometry. To measure surface CAR expression, cells were rinsed with PBS, removed from the plate with Cell Stripper (Mediatech) at room temperature, and washed twice. All washes and incubations were done in 2% FBS in PBS. Cell pellets were resuspended in 50 *μ*L primary CAR antibody (1 : 250, Upstate Biotechnologies, clone RmcB, cat no. 05–644) or isotype antibody (BD Pharmingen mouse IgG1, cat no. 554121) and incubated for 1 h at room temperature. After washing twice, cell pellets were resuspended in secondary Ab (1 : 100, Biomeda Phycoprobe R-PE anti-mouse IgGH+L, cat no. p63) for 30 min at room temperature in the dark. Cells were washed twice prior to flow cytometry analysis. Flow cytometry was performed in the MUSC core facility on a FACSCalibur (Becton Dickenson, Bedford, MA) and data analyzed with the FlowJo version 8.8.6 software.

### 2.7. Animal Experiments and Statistical Analysis

All animal studies were performed under a protocol approved by the Institutional Animal Care and Use Committee of the Medical University of South Carolina. Subconfluent C4-2b cells were trypsinized, pelleted, and resuspended to a concentration of 2 × 10^7^ cells/mL in PBS. A volume of 100 *μ*L (2 × 10^6^ cells) was injected into the flanks of mice. Established tumors were injected with 1 × 10^9^ infectious units (IUs) of adenovirus on day 0, day 3, and day 6. Tumor size was monitored with a digital caliper and volume calculated using the formula: tumor volume = width^2^  × length × 0.52. Animals were sacrificed 14 days after treatment initiation.

 Linear regression was used to estimate the slope (expected change in tumor volume associated with one-day time increase) for each tumor. Group comparisons of the estimated slope values were performed using the Wilcoxon rank-sum test, a nonparametric alternative of the *t*-test, which is robust to deviations from the normality assumption. The *P* values for this test were computed using exact calculations due to the small sample sizes. Analyses have been performed using SAS version 9 (SAS Institute, Cary, NC).

## 3. Results and Discussion

### 3.1. Adenoviral TRAIL Gene Therapy Delays the Growth of Established Castration-Resistant Tumors

**Figure 1:**
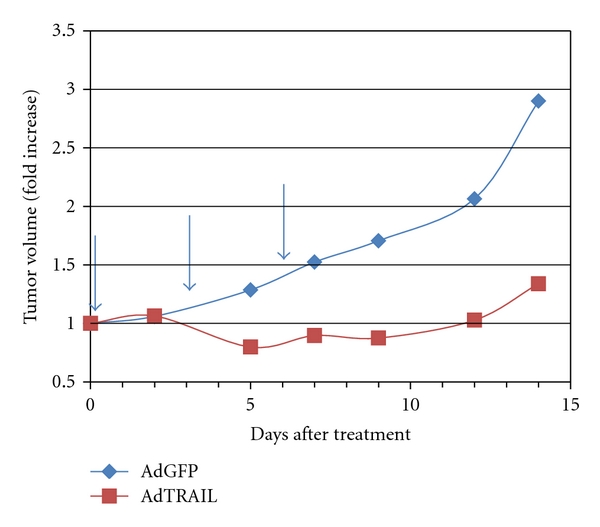
Efficacy of AdTRAIL in subcutaneous C4-2b xenografts. Male mice were subcutaneously injected with 2 million C4-2b cells. After tumor establishment, mice received three intratumoral injections of 1 × 10^9^ IU of AdGFP or AdTRAIL every 3 days (arrows). Data shown are from 6 tumors/group (*P* = 0.015).

Adenoviral gene therapy using TRAIL as the therapeutic transgene has recently entered the clinic. Intraprostatic injection of the AdTRAIL virus was found to be safe, but efficacy remains to be determined [4]. While localized prostate cancer can be effectively managed and/or treated as evidenced by 5-year survival rates approaching 100%, more advanced castration-resistant disease remains incurable. Since novel treatment options for castration-resistant prostate cancer are urgently needed, we evaluated the efficacy of adenoviral TRAIL gene therapy in C4-2b cells, the most aggressive cell line of the LNCaP progression model [5]. The LNCaP progression model mimics phenotypic and genotypic changes observed in human prostate cancer as it progresses from a castration-sensitive to castration-resistant phenotype and has therefore been widely used for *in vitro* and *in vivo* studies of the disease. Previous *in vitro* studies from our laboratory have shown that soluble, recombinant TRAIL has little effect on the viability of C4-2b cells, whereas an adenovirus-expressing membrane TRAIL (AdTRAIL) induced cell death in a concentration-dependent manner [6]. To determine the efficacy of AdTRAIL *in vivo*, we subcutaneously injected 2 million C4-2b cells and initiated treatment with AdGFP or AdTRAIL when tumors were established. As shown in Figure 1, AdTRAIL significantly reduced the rate of tumor growth *in vivo* (*P*-value for the slopes between AdGFP- and AdTRAIL-treated mice was 0.015). Although the results were statistically significant, tumor growth began to recover after treatment termination, and tumor eradication was not achieved in any animal. It is not clear whether expression of TRAIL in our model inhibited tumor growth, which then resumed when transgene expression declined or whether TRAIL induced apoptosis in a subset of cells, while nontransduced cells continued to proliferate. Either effect would result in a delay in tumor outgrowth. A recent study with human prostate cancer tissue found that stromal TRAIL expression is associated with better disease outcome, which supports the idea of controlling tumor outgrowth [7]. On the other hand, it is well known that *in vivo* rates of adenoviral transduction rates are low, and it is likely that only a small fraction of the tumor cells were virally transduced. Thus, adenoviral TRAIL gene therapy would likely be enhanced by combination therapies that improve transduction and/or increase bystander activity of the TRAIL transgene.

### 3.2. Progressive Loss of CAR Surface Expression in the LNCaP Progression Model Is Associated with Reduced Adenoviral Transduction

**Figure 2:**
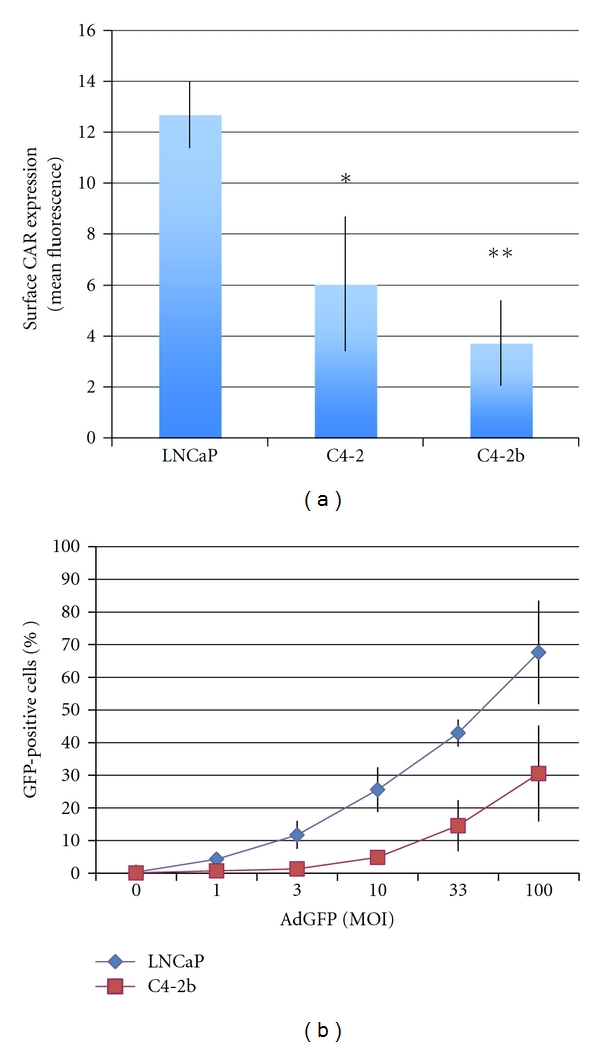
CAR expression and adenoviral infectivity. (a) Surface CAR expression was determined by flow cytometry. Mean fluorescence of cells stained with CAR antibody minus mean fluorescence of cells stained with the isotype control was determined. The average ± SD was calculated, and significance was determined using the student *t*-test. *P < 0.05, **P < 0.0005. (b) Adenoviral infectivity was determined by the expression of GFP 48 hours after transduction. All data shown are the mean ± SD from 3 independently performed experiments.

Efficacy of adenoviral gene therapy depends in part on the ability of the virus to transduce cells. Adenovirus enters cells by binding to a cell surface protein called coxsackie and adenovirus receptor (CAR) [8, 9]. In human prostate tissue, a decrease in CAR expression correlated with increasing tumor grade [10]. We therefore asked whether a decrease in CAR is also observed in the LNCaP progression model. Using flow cytometry to measure surface CAR, we found that compared to LNCaP cells, C4-2 and C4-2b cells had reduced their surface CAR expression by approximately 50% and 70%, respectively (Figure 2(a)). The decrease in surface CAR expression also resulted in poorer adenoviral transduction efficiency. As shown in Figure 2(b), following exposure to 100 MOI, only 30% of C4-2b cells were transduced by adenovirus as determined by GFP transgene expression. In LNCaP cells, this level of infectivity was achieved at approximately 10 MOI, indicating that a 10-fold higher concentration of adenovirus was required to transduce C4-2b cells. These results demonstrate that the decrease in CAR expression observed in human prostate tumors also occurs in the LNCaP progression model. Although CAR was originally discovered as a viral receptor, it has subsequently been identified as an adhesion protein [11]. Thus, the decrease in CAR expression in parallel with increasing tumorigenicity may at least in part contribute to the metastatic properties of C4-2 and C4-2b cell lines, since a decrease in adhesion is a prerequisite for dissemination of tumor cells to distant sites.

### 3.3. Treatment with Histone Deacetylase Inhibitors Restores Surface CAR Expression and Enhances Adenoviral Transduction Efficiency as well as TRAIL-Mediated Caspase Activity

**Figure 3:**
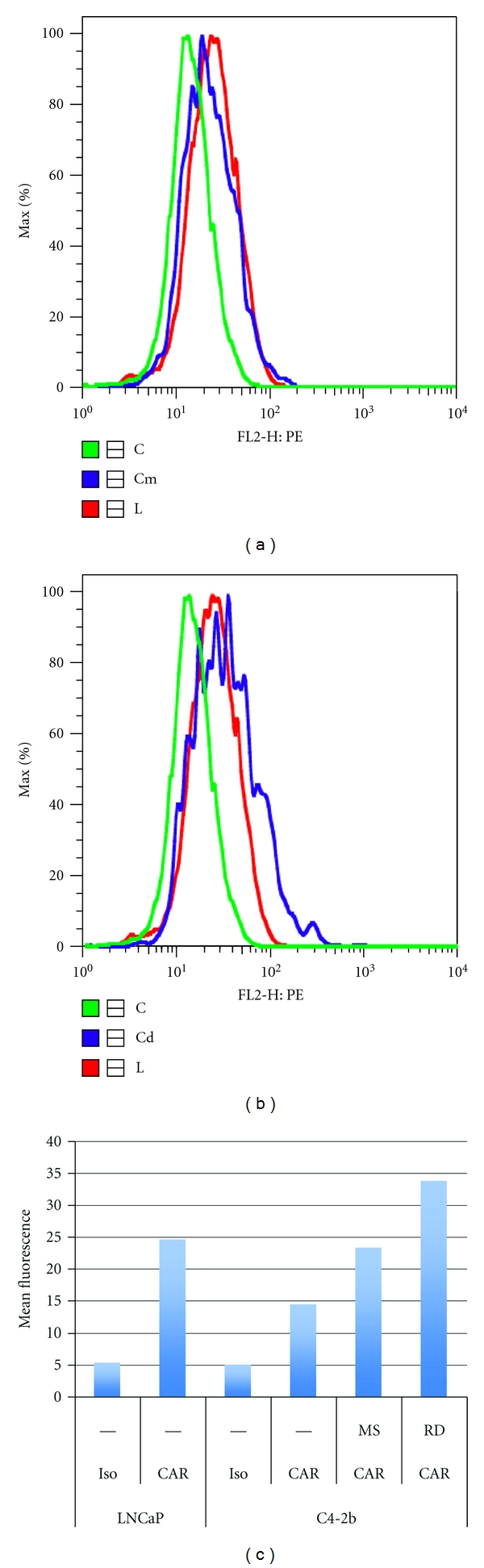
The effect of HDACi on surface CAR expression in C4-2b cells. Surface CAR expression was determined by flow cytometry in LNCaP cells (L = red lines), C4-2b cells (C = green lines), and C4-2b cells in the presence of HDACi (Cd or Cm = blue lines). Cd indicates that 100 ng/mL romidepsin was used, whereas Cm indicates a dose of 1 *μ*M MS-275. (c) Graphical representation of the data. RD = romidepsin.

The decrease of CAR expression has previously been shown to occur by epigenetic modulation of the CAR gene promoter [12]. Inhibition of histone deacetylation but not DNA methylation restored CAR expression in several urogenital cancer cell lines [12]. Numerous histone deacetylase inhibitors (HDACiS) are being explored in clinical trials as single agents or in combination therapies [13]. These chemotherapeutic agents function, at least in part, to reverse epigenetic changes that contribute to the development and progression of malignancies. We have previously shown that the HDACi romidepsin and MS-275, which are both under investigation for their therapeutic potential against prostate cancer, can selectively enhance AdTRAIL gene therapy in castration-sensitive LNCaP cells and that an increase in CAR expression contributed to this effect [3]. We therefore next asked how HDACi affect CAR expression and adenoviral infectivity in the more aggressive, castration-resistant cell line C4-2b. As shown in Figure 3, both romidepsin and MS-275 enhance surface CAR expression in C4-2b cells to levels that were comparable to parental LNCaP cells.

**Figure 4:**
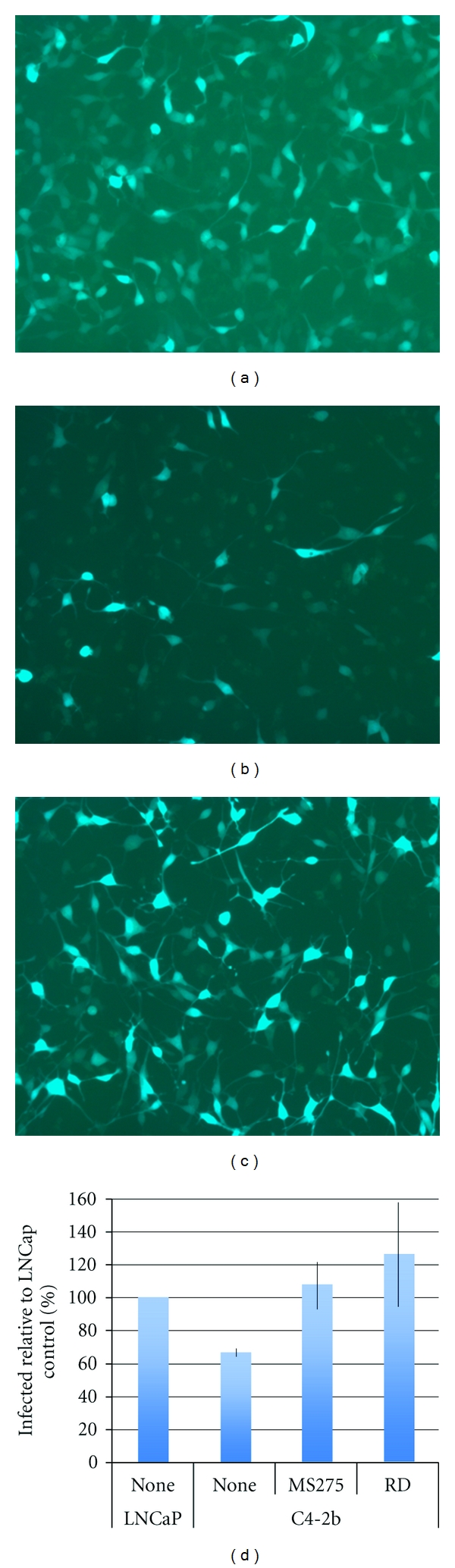
Analysis of GFP expression by fluorescence microscopy and quantification by flow cytometry. LNCaP (a) and C4-2b (b, c) were plated overnight and exposed to 10 MOI AdGFP in the absence (a, b) or presence of (c) 10 *μ*M MS-275. Fluorescent images were collected using identical camera settings 48 hrs after viral transduction (magnification = 200x). (d) Quantification of GFP-positive cells by flow cytometry 24 hrs after viral transduction in the absence or presence of 100 ng/mL romidepsin or 10 *μ*M MS-275. Data shown are the mean ± SD from three experiments.

**Figure 5:**
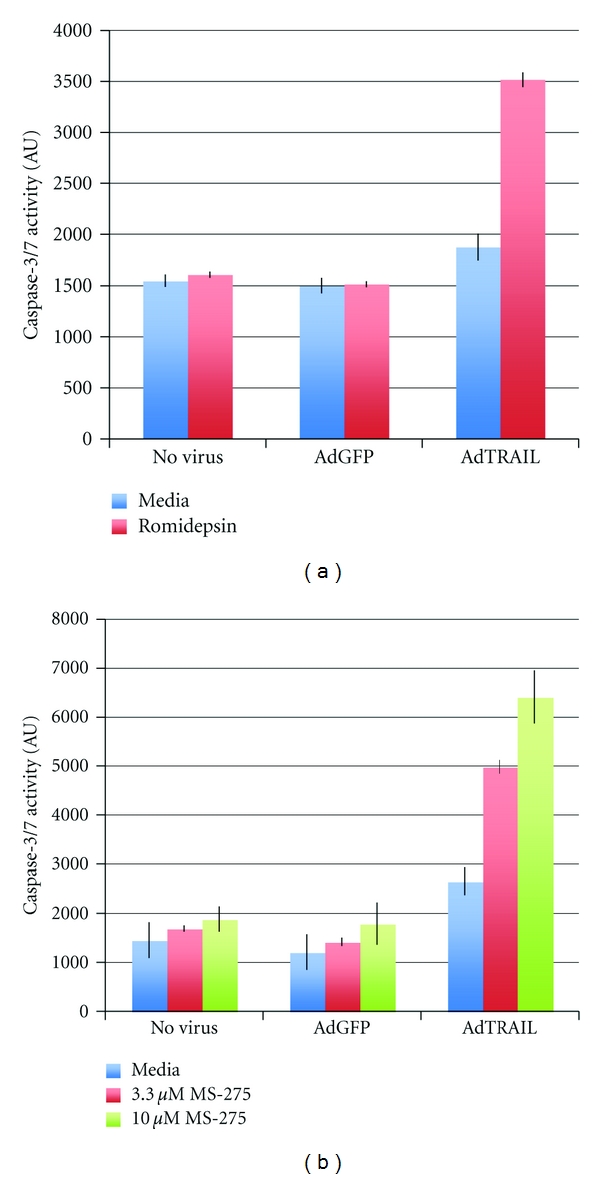
Caspase-3/7 activity in C4-2b cells. C4-2b cells were left uninfected or were transduced with 10 MOI of AdGFP or AdTRAIL in the absence or presence of MS-275 (b) or 10 ng/mL romidepsin (a). Caspase-3/7 activity was determined at 48 hours. Data shown are the mean ± SD of an assay performed in triplicate.

Next, we investigated the biological consequences of HDACi-mediated restoration of CAR expression. As shown in Figure 4, treatment with HDACi increased adenoviral infectivity in C4-2b cells to levels that were comparable to those in LNCaP cells. We also investigated the effect of HDACi on caspase-3/7 activation following transduction with AdTRAIL. As shown in Figure 5, although AdTRAIL alone did result in increased caspase-3/7 activity compared to untreated or control-infected (AdGFP) C4-2b cells, the effect was greatly enhanced by treatment with HDACi.

Restoration of CAR expression by HDACi could improve the efficacy of gene therapy not only by increasing uptake of adenovirus during the initial delivery but also by promoting viral spread throughout the tumor when conditionally replicative adenovirus is used. In addition, HDACi have been shown to synergize with TRAIL; thus, in addition to improved adenoviral transgene expression, a higher percentage of tumor cells may be killed due to bystander activity. Lastly, by increasing CAR expression, cancer cells may become more adhesive and less prone to metastasize to distant sites.

### 3.4. Histone Deacetylase Inhibitors Enhance CMV-Mediated Gene Expression

**Figure 6:**
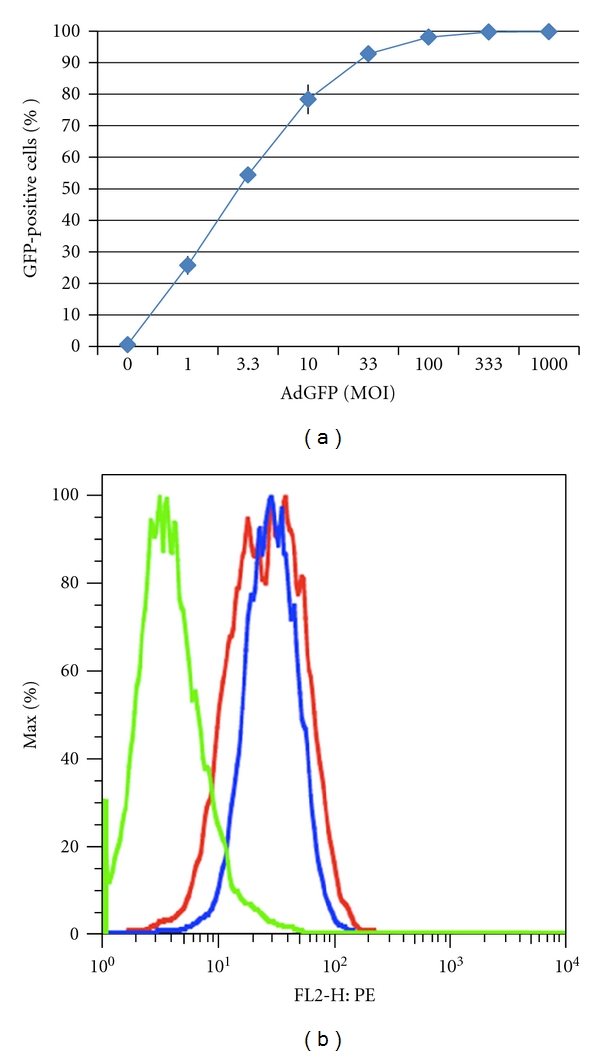
Adenoviral infectivity and surface expression in 22Rv1 cells. (a) 22Rv1 cells were transduced with increasing amounts of AdGFP. The presence of GFP-positive cells was quantified by flow cytometry at 48 hrs after transduction. Data shown are the mean ± SD of an assay performed in duplicate. (b) Surface CAR expression was determined by flow cytometry (green = isotype, blue = anti-CAR, untreated cells, and red = anti-CAR in cells treated with 10  *μ*M MS-275).

**Figure 7:**
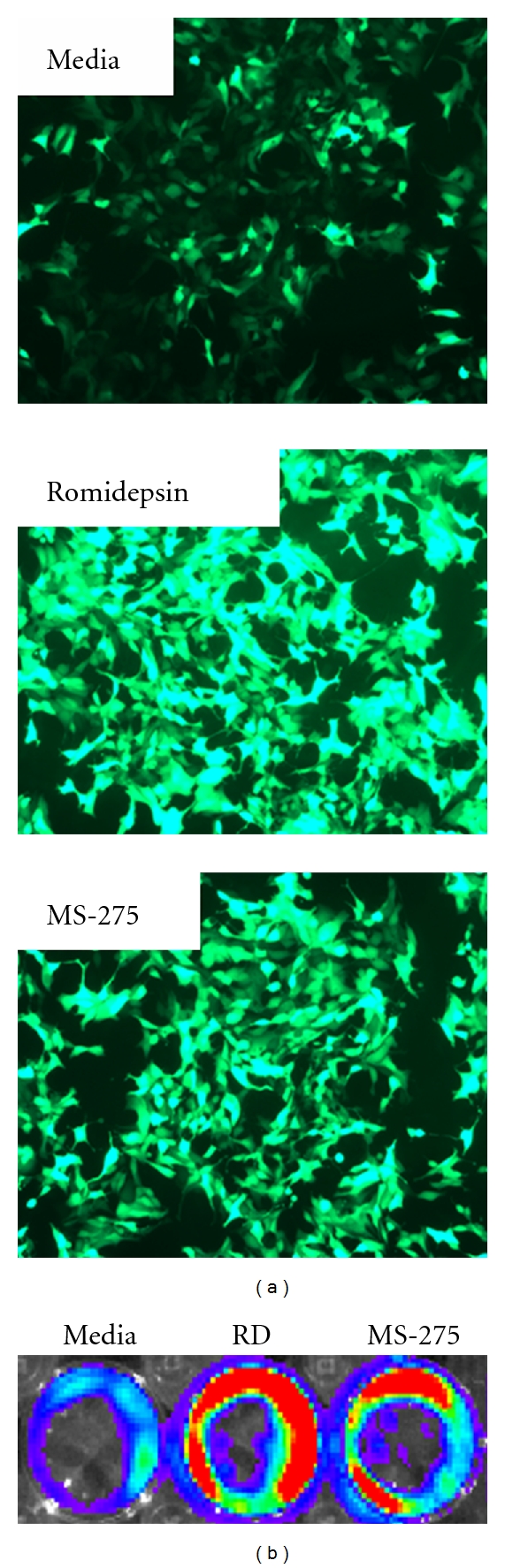
The effect of HDACi on gene expression in 22Rv1 cells. (a) 22Rv1 cells were transduced with 10 multiplicities of infection with AdGFP in the absence or presence of 250 ng/mL Romidepsin or 1 *μ*M MS-275. Fluorescent images were collected 24 hrs after viral transduction (magnification = 200x). (b) 22Rv1 cells were transfected with pCMV.luc. The luciferase signal was quantified 48 hrs after transfection in the absence or presence of 100 ng/mL romidepsin (RD) or 1 *μ*M MS-275. The photograph is a false color image of three representative wells indicating relative numbers of photons produced during a timed interval after luciferin addition. Red indicates more photons; blue indicates less photons.

**Figure 8:**
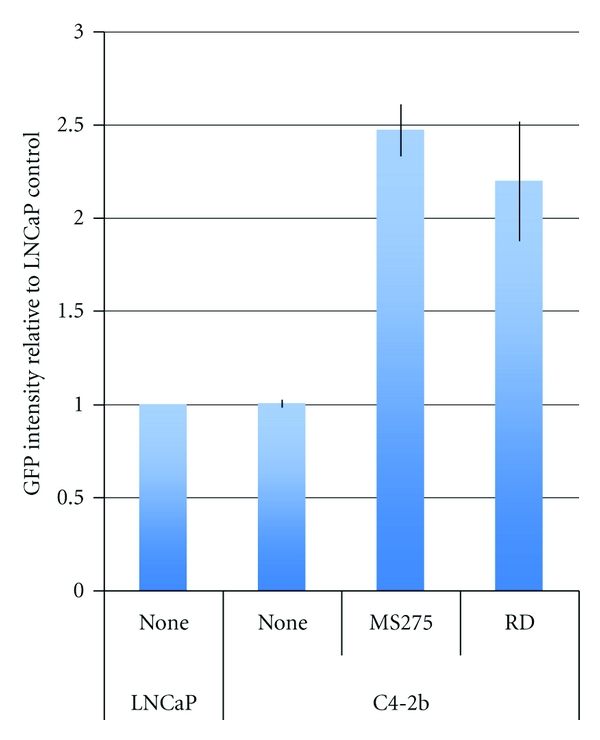
The effect of HDACi on GFP intensity in C4-2b cells. Cells were plated overnight and exposed to 10 MOI AdGFP in the presence or absence of 100 ng/mL Romidepsin or 10 *μ*M MS-275. The geometric mean fluorescence intensity within GFP-positive cells is expressed as a fold change relative to LNCaP cells. Data shown are the Mean ± SD from three experiments.

Next, we asked whether the increased efficacy of HDACi treatment on adenoviral GFP transgene expression was solely due to increased CAR expression. To address this question, we chose 22Rv1 cells, which are highly tumorigenic but nonmetastatic [14]. These cells express high levels of surface CAR (mean fluorescence CAR antibody minus mean fluorescence isotype control antibody: 24.20 ± 5.76, *n* = 4) and are easily transduced with adenovirus (Figure 6(a)). Interestingly, while HDACi did not further increase surface CAR expression (Figure 6(b)), adenoviral transgene expression was strongly enhanced as shown by fluorescent microscopy of GFP levels (Figure 7(a)). Since GFP expression in the adenoviral vector is under control of the CMV promoter, we wondered whether HDACi enhanced promoter activity. To determine the effect of HDACi on CMV promoter activity, we transfected a CMV-driven luciferase reporter gene into 22Rv1 cells and measured luminescence. Our results indicated that both romidepsin and MS-275 enhance luciferase expression from the CMV promoter (Figure 7(b)). Similar results were obtained in C4-2b cells (data not shown). We also analyzed the GFP intensity in LNCaP and C4-2b cells following transduction with AdGFP. Although C4-2b cells are not as readily transduced with adenovirus (see Figure 2(b)), the intensity of GFP expression among GFP-positive cells was similar to LNCaP cells (Figure 8). HDACi significantly enhanced GFP intensity, suggesting that this effect may be due to stimulation of the CMV promoter and thus GFP transgene expression (Figure 8). In agreement with our results, VanOosten et al. previously reported that romidepsin enhanced CMV promoter activity in Du145 prostate cancer cells [15]. HDACi-mediated enhancement of transgene expression from the CMV promoter would be beneficial not only for viral gene therapy but also for nonviral approaches. For example, recent studies demonstrated that HDACi enhanced the efficacy of a CMV-driven DNA vaccine and may also improve polymer-mediated gene delivery [16, 17]. Interestingly, HDACi inhibited gene expression from a prostate-specific promoter, indicating that combination of these drugs with tissue-specific promoters needs to be carefully evaluated [18]. In cases when HDACi inhibit tissue-specific promoter activity, the human telomerase promoter, which has been used for transcriptional targeting of transformed cells, may be a suitable choice [19].

## 4. Conclusions

We propose that maximum efficacy against advanced prostate cancer can be safely achieved by combining HDACi with TRAIL gene therapy using conditionally replicative adenovirus (CRAd). CRAd vectors propagate under certain conditions subsequently killing the infected cell and infecting neighboring cells. The resulting lateral spread of the virus is thought to increase the efficacy of adenoviral gene therapy. However, lateral spread to malignant cells would be impaired if surface CAR expression has been epigenetically silenced. Thus, HDACi therapy when used in combination with CRAd TRAIL gene therapy can be envisioned to (1) restore surface CAR levels and facilitate viral transduction and spread among cells with previously reduced CAR expression, (2) through positive influence on CMV promoter activity enhance TRAIL transgene expression, and (3) by negative influence of genes associated with apoptosis resistance would boost bystander activity of the TRAIL transgene. An additional benefit of HDACi therapy would be that the restoration of CAR as well as other epigenetically silenced genes such as E-cadherin that is involved in maintenance of adhesion may decrease the risk of metastasis. An important next step will be to evaluate HDACi/AdTRAIL combination therapy in an *in vivo* model. Based on the effects observed *in vitro*, it is likely that combination therapy results in increased efficacy, yet due to the pleiotropic effects of HDACi, a primary mechanism of action may be difficult to discern. With regard to prostate cancer, it would be interesting to evaluate systemic HDACi therapy in combination with intraprostatic injections of AdTRAIL in orthotopically implanted C4-2b cells to determine whether this therapeutic strategy has the potential to diminish dissemination of advanced, castration-resistant prostate cancer.

## 5. Main Take-Home Message

Histone deacetylase inhibitors have multiple beneficial effects including improved adenoviral gene therapy by restoration of CAR expression on aggressive tumor cells and increased transgene expression from the CMV promoter, which may enhance not only viral but also nonviral gene therapy.
